# Bembidion (?Nipponobembidion) ruruy sp. n., a new brachypterous ground beetle (Coleoptera, Carabidae) from Kunashir Island, Kuriles, Russia

**DOI:** 10.3897/zookeys.463.8504

**Published:** 2014-12-12

**Authors:** Kirill V. Makarov, Yuri N. Sundukov

**Affiliations:** 1 Department of Zoology and Ecology, Moscow State Pedagogical University, 129164 Moscow, Russia; 2 State Nature Reserve “Kurilskiy”, 694500 Yuzhno-Kurilsk, Sakhalinskaya oblast’, Russia

**Keywords:** *Bembidion*, Bembidiini, Trechitae, morphology, taxonomy, relict, Kuril Archipelago, volcano, faunogenesis

## Abstract

A new species, Bembidion (?Nipponobembidion) ruruy **sp. n.**, is described from the foot of Ruruy Volcano, Kunashir Island, Kuril Archipelago, Russia. It is only the second consubgener, being characterized by the reduced wings, the rounded elytral shoulders, and the backward position of the posterior supra-orbital pore. In this connection, the subgenus *Nipponobembidion* Habu & Baba, 1968 is rediagnosed and both of its species are keyed. It might have originated from *Plataphodes* Ganglbauer, 1891, possibly in relation to volcanic activities in the region.

## Introduction

The carabid fauna of Kunashir Island, Kuriles, Russia, can be considered as well-known (see review by Lafer 2002), comprising, according to our data, at least 150 species. Despite the small size of the island and the narrow straits separating it from the neighbouring islands, there are several subendemic taxa. The genus *Bembidion* Latreille, 1802 alone, based on our data, is represented on Kunashir by 23 species, none of which has hitherto been regarded as endemic.

In the summer of 2013, a small series of a remarkable brachypterous *Bembidion* were collectedin the northern part of Kunashir Island, and description is provided below.

## Materials and methods

Standard methods were applied for treating the material. Genitalia were mounted on permanent slides using the Faure-Berlese medium. External characters were studied with the help of MBS-1 and Leica M165C stereoscopes, the genitalia examined under MBR-15 and Micromed 2, version 2–20 compound microscopes. Pictures were taken using a Canon EOS 5D Mark III camera with a Canon MP-E 65 mm objective lens and a Canon PowerShot A640, while the extended focus images by means of the Zerene Stacker software.

The abbreviations used in text are as follows:

EL greatest length of elytra;

EW greatest width of elytra;

HL length of head, measured along the median line from fore margin of clypeus to rear edge of the temples;

HW greatest width of head;

L body length from fore margin of clypeus to elytral apex;

Ls sum of Hl, Pl(t) and EL;

PA width of pronotal apex;

PB width of pronotal base;

PL(m) length of pronotum, measured along the median line;

PL(t) greatest length of pronotum;

PW greatest width of pronotum;

M arithmetic mean.

Designations of the sclerotized parts of the aedeagus are given after Maddison (1985). Female reproductive tract characters follow the terminology of Liebherr and Will (1998). Abbreviations given in the illustrations are the following:

AG apical gonocoxite;

BC bursa copulatrix;

BS brush sclerite;

DP dorsal fig;

FL flagellum;

FS flagellar sheath;

LD latero-distal sclerite;

OP ostial microtrichial patch;

RL right lobe of central sclerite complex;

SD spermathecal duct;

SG spermathecal gland;

SP spermatheca;

VSP ventral sclerite patch.

The material has been shared between the collections of the Zoological Institute, Russian Academy of Sciences, St. Petersburg (ZISP) and the Moscow State Pedagogical University (MSPU), as well as in the private collection of the second author, kept in Lazo, Maritime Province, Russia (CSLR).

## Taxonomy

### Bembidion (?Nipponobembidion) ruruy sp. n.

Taxon classificationAnimaliaColeopteraCarabidae

http://zoobank.org/8548C873-D251-4CA8-A647-D76B4DF0521C

**Figure 1.**
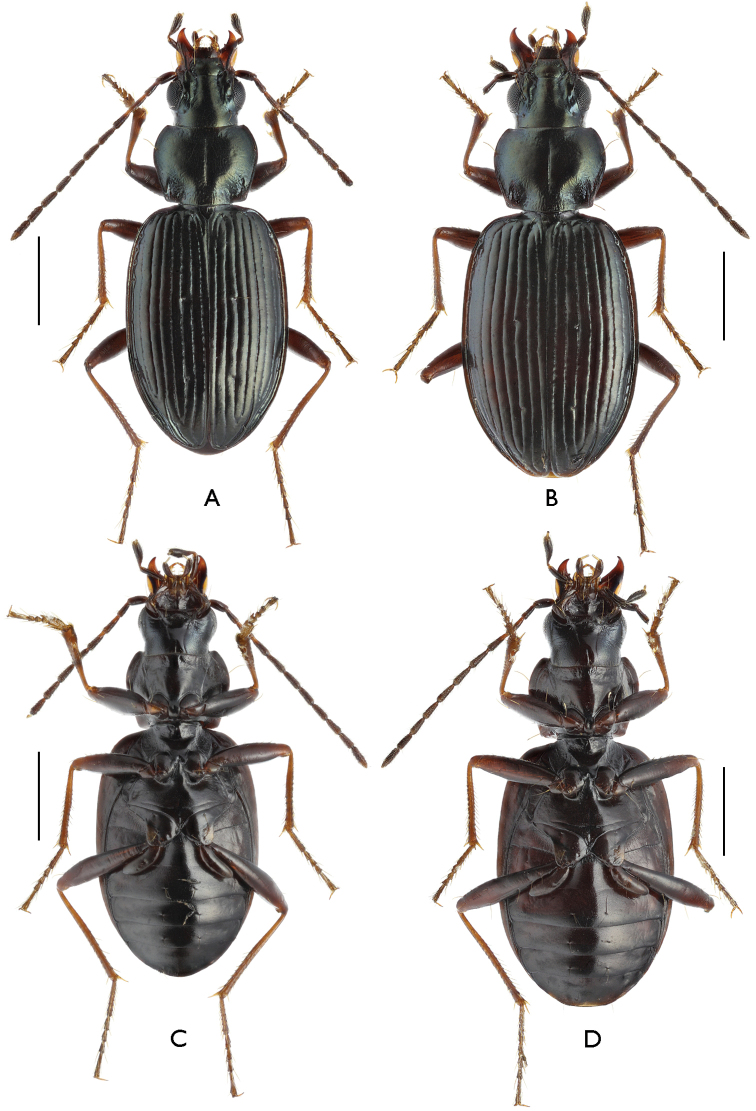
Habitus of Bembidion (?Nipponobembidion) ruruy sp. n., dorsal (**A, B**) and ventral (**C, D**) views. Scale bars 1.0 mm. **A, C** male, holotype **B, D** female, paratype.

**Figure 2.**
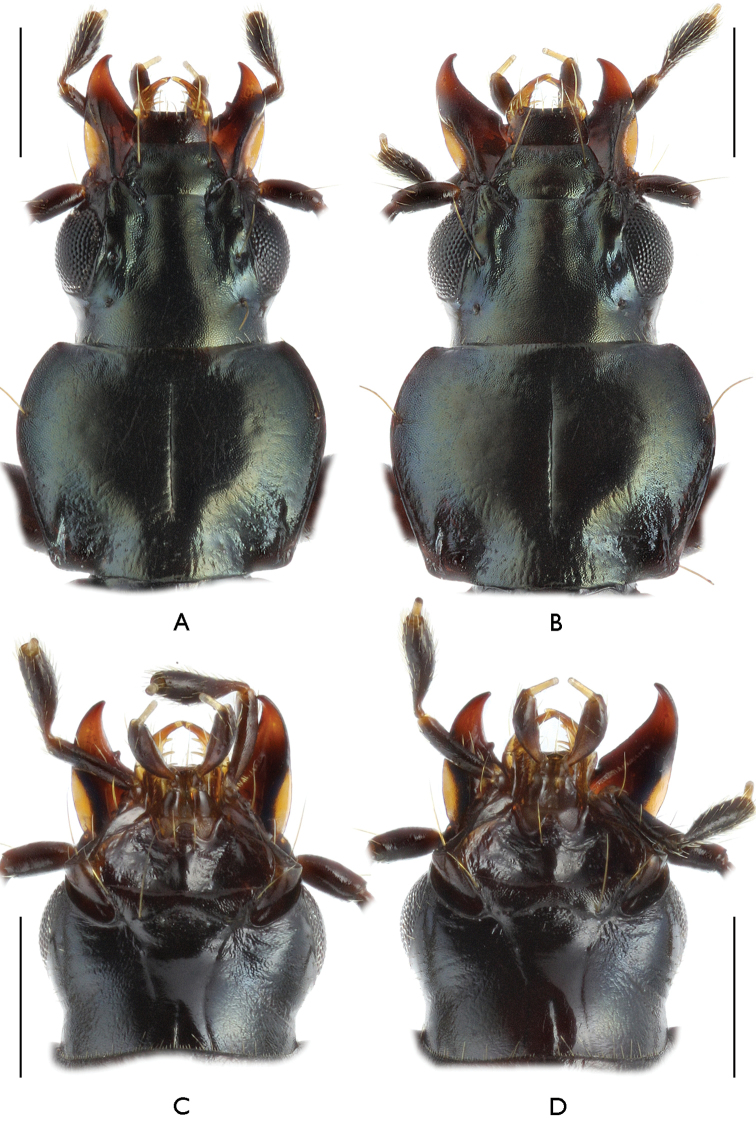
Head and pronotum of Bembidion (?Nipponobembidion) ruruy sp. n., dorsal (**A, B**) and ventral (**C, D**) view. Scale bars 0.5 mm. **A, C** male, holotype **B, D** female, paratype.

**Figure 3.**
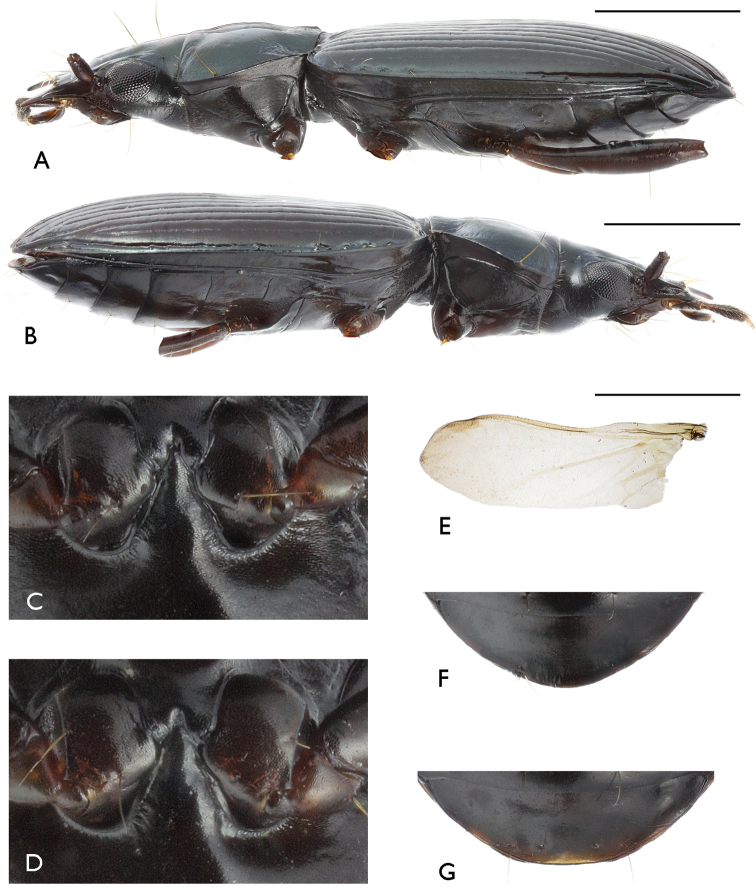
Bembidion (?Nipponobembidion) ruruy sp. n. Scale bars 1.0 mm. **A, C, E, F** male, holotype **B, D, G** female, paratype **A, B** body, lateral view (extremities not shown) **C, D** metacoxae and metepisternal process, ventral view **E** hindwing **F, G** anal sternite, ventral view.

**Figure 4.**
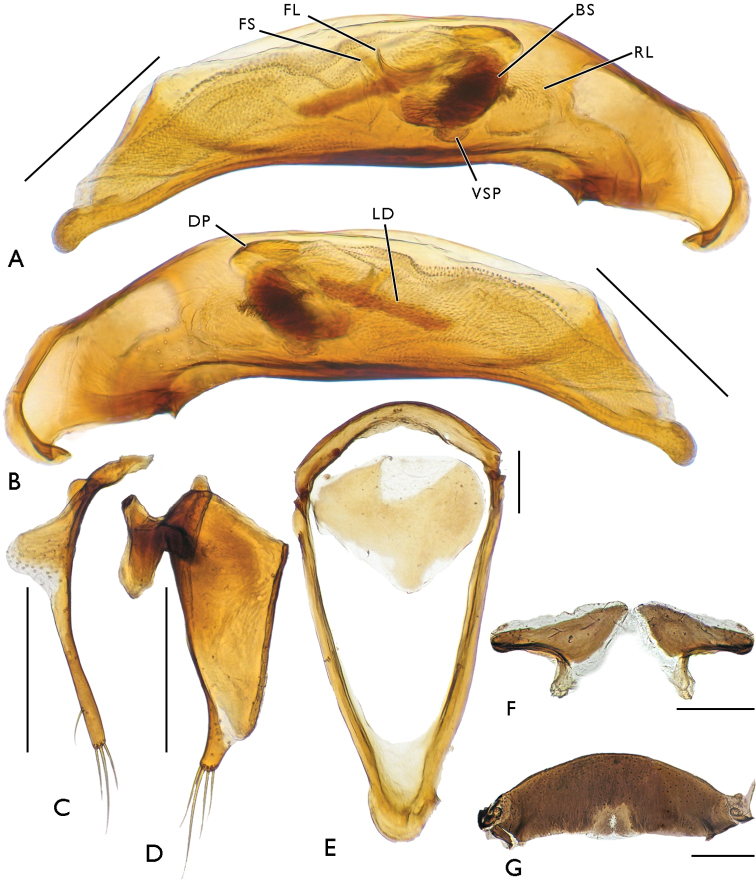
Male genital apparatus of Bembidion (?Nipponobembidion) ruruy sp. n., holotype. Scale bars 0.25 mm. **A** aedeagus, right side **B** aedeagus, left side **C** right paramere **D** left paramere **E** sternite 9, ventral view **F** sternite 8 **G** tergite 8.

**Figure 5.**
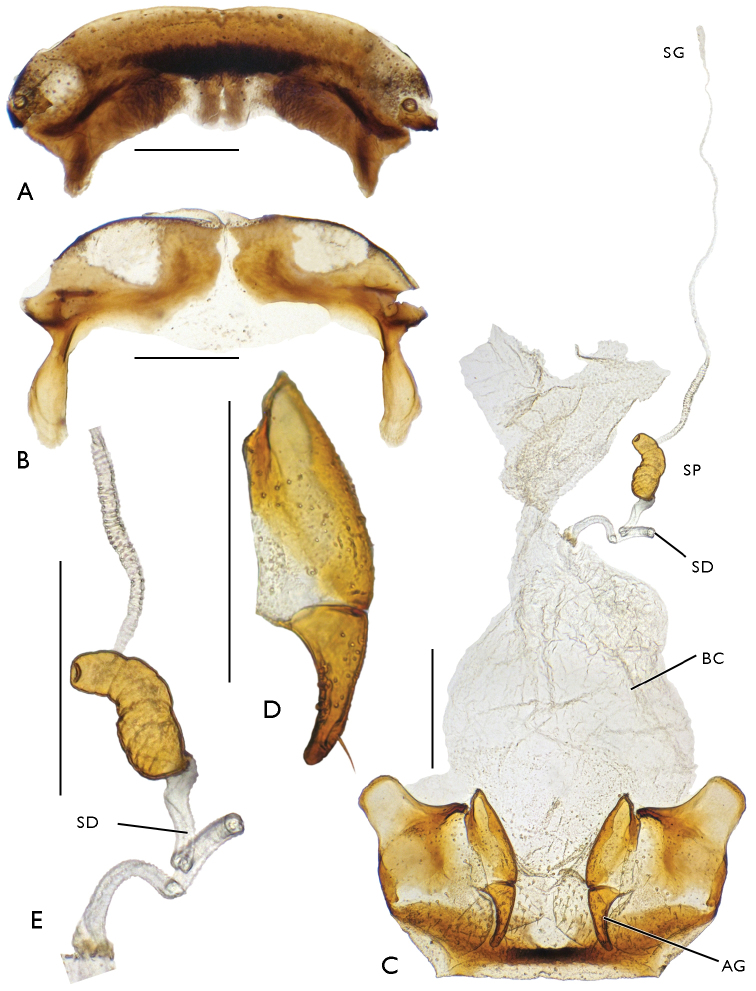
Female genital apparatus of Bembidion (?Nipponobembidion) ruruy sp. n., paratype. Scale bars 0.25 mm. **A** tergite 8 **B** sternite 8. **C** genital apparatus **D** left gonocoxite **E** spermatheca.

Figs 1 , 2 , 3 , 4 , 5

#### Holotype

♂ with labels: “Northern Kunashir, 2.5 km NW of Cape Nelyudimyi, N44°29.433', E146°11.783', 4.VIII.2013, leg. K. Makarov & Y. Sundukov” and “HOLOTYPE Bembidion (Nipponobembidion) ruruy Makarov & Sundukov 2014 [printed on red paper]”.

The specimen is deposited in ZISP, the genitalia mounted on a slide in Faure-Berlese medium are pinned beneath.

#### Paratypes.

4 ♂, 3 ♀ from northern Kunashir, 2.5 km NW of Cape Nelyudimyi, N44°29.433', E146°11.783', 4.VIII.2013, leg. K. Makarov & Y. Sundukov; [1 ♀ — ZISP, 1 ♂, 1 ♀ — MPSU, 3 ♂, 1 ♀ — CSRL]; 1 ♂ from northern Kunashir, mouth of Dokuchaev River, N44° 30.317', E146°10.800', 30.VII.2013, leg. Y. Sundukov [CSLR].

#### Type locality.

RUSSIA: Kuril Archipelago: Kunashir Island, at the foot of Ruruy Volcano.

#### Etymology.

The species epithet is a Latinized noun in apposition to reflect the name of the volcano at the foot of which the new species was found.

#### Description.

Body faintly convex. Length 4.1–5.1 mm, width 1.4–2.1 mm.

Head and pronotum black, elytra black- or dark brown, entire upperside with a faint bronze or bluish lustre. Head appendages: antennae black-brown with lighter bases of antennomeres 2–4; palpi black with yellowish apical palpomeres; mandibles entirely brown or with lighter apices; labrum dark brown. Underside black or dark brown. Legs black or black-brown with lighter pro- and mesotrochantera, as well apical parts of metatarsi; all tibiae and tarsi brown (Fig. 1).

Upperside devoid of punctation, only basal pits of pronotum with a few small punctures at bottom. Dorsal microsculpture of head, including clypeus and labrum, with rough isodiametric meshes especially rough inside frontal furrows. Disc of pronotum finely microsculptured, with transverse meshes growing isodiametric and rougher like on head towards margins (Fig. 2A, B). Elytra with a very delicate, transverse microsculpture; the latter near basal elytral margin isodiametric and rougher. Head appendages and extremities with a distinct microsculpture of broad meshes. Microsculpture of pleurites and sternites isodiametric, strongly obliterated at edges of abdominal sternites.

Standard dimensions (mm): HW 0.78–0.95 (M 0.87); HL 0.58–0.74 (M 0.62); PA 0.75–0.94 (M 0.84); PW 1.03–1.30 (M 1.16); PB 0.80–0.95 (M 0.89); PL(t) 0.88–0.99 (M 0.92); PL(m) 0.83–0.95 (M 0.87); EW 1.60–2.05 (M 1.86); EL 2.38–3.05 (M 2.64); Ls 3.84–4.90 (M 4.18); L 4.10–5.10 (M 4.6).

Head not flattened, together with eyes 1.29–1.42 times as broad as long. Eyes moderately convex. Antennae long, in ♂ as long as elytra, in ♀ 0.90–0.95 their length. Mandibles small, with pointed apices. Labrum trapeziform, with six setae along fore margin. Clypeus trapeziform, with two long setae in fore angles. Tooth of mentum large, edged, broadly rounded at apex; mentum with two setae at base. Submentum with two pairs of large setae, external ones being shorter. Gula in basal 1/3 with a deep, thin, longitudinal sulcus (Fig. 2C, D). Temples short, about 1/3 as long as eye diameter. Two supra-ocular setae: one near the middle of eye, the other behind its caudal margin. Frontal furrows non-diverging, parallel, broad, groove-like, expressed from caudal eye margin nearly up to setae on clypeus. Space between frontal furrow and lateral edge of frons convex, not keel-shaped.

Pronotum (Fig. 2A, B) moderately convex, faintly cordiform, transverse (PW/PL(t) = 1.24–1.33 (M 1.29), PW/PL(m) = 1.28–1.37 (M 1.31), broader than head (PW/HW = 1.31–1.37 (M 1.34), broadest at 3/5 off base. Front margin slightly concave, its fine edging broadly interrupted in the middle. Fore angles faintly produced forward, their apices narrowly rounded. Base faintly convex, at basal pits slightly concave and vaguely edged, a little broader than fore margin (PB/PA = 1.01–1.10, M 1.06). Hind angles not produced, obtuse, slightly skewed forward, rounded at apices. Lateral sides in anterior half moderately convex and regularly rounded, in posterior half rectilinearly narrowed towards hind angles. Lateral margins narrowly edged. Two lateral setae on each side: one in front of maximal breadth, the other at caudal angle. Transverse impressions faint. Basal fovea rather large, roundly triangular, flattened and moderately rugose inside, delimited outside by a faint keel.

Elytra oblong-oval, rather large, broad (EL/EW = 1.39–1.69 (M 1.42), EL/PL(t) = 2.70–3.11 (M 2.91), EW/PW = 1.55–1.67 (M 1.59), slightly convex, broadest at 2/3 of elytral length. Shoulder not protruding, broadly rounded. Basal margin reaching apex of 4^th^ (4 specimens) or 5^th^ (5 specimens) stria, near shoulder arcuated and gradually turning into lateral margin. Lateral margin flattened and narrow. External apical angle broadly rounded. Striae complete, moderately deep, non-punctate, interspaces faintly convex; stria 7 either well-developed or superficial and faint in anterior half, but evident throughout; stria 8 fused to 9^th^ about midway of series umbilicata (Fig. 3A, B). Subscutellar stria rather short, placed inside interspace 1. Subscutellar pore placed at junction of striae 1 and 2. Interval 3 with two well-expressed discal pores lying at stria 3: frontal at ca 2/5, caudal at ca 2/7, off base. Apical stria deep, cariniform, uninterrupted, fused to 5^th^ stria, carrying two setigerous pores: one at apex, the other opposite stria 4. Series umbilicata consisting of eight setae: four in humeral group, and two each in the middle and at apex (Figure 3A, B).

Hindwings (Fig. 3E) shortened, 0.65–0.54 times as long as elytra. Among longitudinal veins only larger ones retained, basal parts of *R*, *CuA*, *CuP*, *AA*_3_ and *AP*_1+2_ being well-expressed while *MP* poorly visible. Transverse veins represented by *r*1 and *r*2, both only incompletely delimiting an *rc* cell (designations after Fedorenko 2009).

Legs moderately long, slender; metatarsus subequal in length to metatibia. All tibiae and meso- and metatarsomeres 1 with distinct longitudinal furrows. Meso- and metatarsomeres 3 and 4 with short dorsal keels. Claws thin, about 0.6 times as long as last tarsomere. Meso- and metafemora with 4 setae near caudal margin. Ventro-apical setae of penultimate tarsomere long, reaching beyond 2/3 of claw length.

Underside non-punctate (Fig. 1C, D). Metasternal process narrowly bordered only on sides (Fig. 3C, D). Metepisterna shortened: external margin 1.5 times as long as breadth along fore margin (1 ♂ and 1 ♀ measured). Metacoxae with three setae. Metatrochantera with one seta midway. Abdominal sternites simple, with neither pubescence nor additional setae; apical sternite at apex with two setae in ♂, four setae in ♀ (Fig. 3F, G).

Aedeagus rather slender, its ventral margin faintly curved, apex moderately broad, rounded. Central sclerite complex (CSC) of endophallus with a large brush sclerite and a rather short, S-shaped flagellum with an adjacent flagellar sheath. Right lobe of CSC poorly sclerotized, distinctly microsculptured, reaching down to basal notch of aedeagus. Left lobe small, sclerotized, devoid of a marked sculpture. Ventral sclerite patch small, lying beneath bush sclerite. Ostial microtrichial patch well-developed. Left part of endophallus with two additional structures: a short row of spinicles located level to brush sclerite and a rather long laterodistal sclerite formed by fused cuticular scales (Fig. 4A, B). Left paramere broad (Fig. 4D), with four (one long in the middle and three short) setae at apex; right paramere (Fig. 4C) narrow, with three (one long in the middle and two short) apical setae and one short subapical seta on ventral side.

Female genital apparatus as in Fig. 5. Apical segment of gonocoxite with 1–2 long, subapical, external setae and 2–3 ensiform setae in basal half of external margin (Fig. 5D). Copulatory bursa oval, without additional sclerites, about 1.5 times as long as broad. Duct of spermatheca long, fluted, terminating inside copulatory bursa ventrolaterally. Spermatheca (Fig. 5E) well-sclerotized, elongated and clearly curved, 1.5–2.0 times as broad as duct. Glandular duct long, entering inside distal part of spermatheca.

#### Distribution.

Kunashir Island, southern Kuriles, Russia; known only from the island’s northern part at the foot of Ruruy Volcano.

#### Habitats.

**Figure 6.**
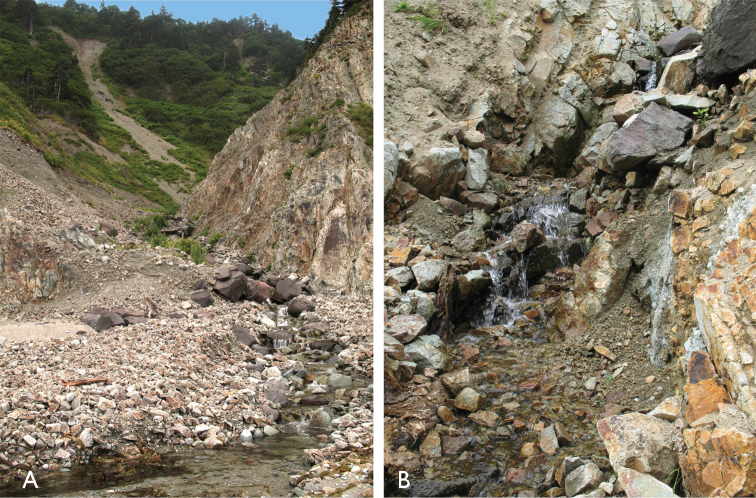
Habitat of Bembidion (?Nipponobembidion) ruruy sp. n., northern Kunashir, 2.5 km NW of Cape Nelyudimyi (N44°29.433', E146°11.783').

Much of the type series was taken inside a water-logged gravel-clay mixture and in crevices of a wet rocky cliff at the bank of a mountain stream running through a narrow mudslide-formed canyon (Fig. 6). The same places yielded abundant Bembidion (Plataphodes) tetraporum Bates, 1883, Bembidion (Ocydromus) dolorosum (Motschulsky, 1850) and Nebria (Nakanebria) shibanaii Uéno, 1955.

#### Comparative notes.

The shape of the head and pronotum, the elytral chaetotaxy pattern, and the complex of characters related to flight loss (shortened elytra, obliterated elytral shoulder, short metepisterna) allow for *Bembidion ruruy* sp. n. to be assigned to the hitherto monobasic Japanese subgenus *Nipponobembidion* Habu & Baba, 1968. However, the new taxon differs significantly from *Bembidion ainu* Habu & Baba, 1968, the type species of that subgenus, by its habitus, endophallus armament, and perhaps also its paramere chaetotaxy, the left paramere in the sole male *Bembidion ainu* known to date carrying only one apical seta. The following key can be proposed.

### Key to the species of *Nipponobembidion* Habu & Baba, 1968

**Table:** 

1	Larger (4–5 mm), upperside monochromous, black or brown with metallic lustre; legs and antennae monochromous, dark brown. Metepisterna elongated, external margin 1.5 times as long as breadth along fore margin. Metasternal process not edged at apex. Kunashir, Ruruy Volcano	***Bembidion ruruy* sp. n.**
–	Smaller (3–3.5 mm), head and pronotum black, elytra brown to red-brown; antennomere 1 and legs light, reddish. Metepisterna very short, external margin only 1.12 times as long as breadth along fore margin. Metasternal process entirely edged. Hokkaido, Daisetsu Volcano	***Bembidion ainu* Habu & Baba**

## Discussion

In spite of the considerable similarity between *Bembidion ainu* and *Bembidion ruruy* sp. n. related to wing reduction, as well as the chaetotaxy pattern peculiarities, these species differ by a number of significant characters. Furthermore, the metasternal process in *Bembidion ainu* is entirely edged, the basal edging of the elytra only reaching the 4^th^ stria while the endophallus armament is different.

The presence or absence of an edging on the anterior process of the metathorax is traditionally significant when taxonomically sorting out the *Plataphus*-complex (*Plataphus* Motschulsky, 1864, *Plataphodes* Ganglbauer, 1891, *Trichoplataphus* Netolitzky, 1914, *Blepharoplataphus* Netolitzky, 1920) and the *Ocydromus*-complex (*Ocydromus* Clairville, 1806, *Peryphus* Dejean, 1821, *Asioperyphus* Vysoký, 1986 and allied groups). Based on the above distinctions, *Bembidion ainu* and *Bembidion ruruy* sp. n. could be treated as belonging to different species groups of *Bembidion*, suggesting parallel developments related to wing reduction.

It is noteworthy, however, that the significance of that character has repeatedly been questioned. Thus, in the recent review of the phylogeny of *Bembidion* by Maddison (2012), the subgenera *Plataphus* and *Plataphodes*, as well as the “*simplex*” and “*kuprianovi*” species groups (in the sense of Lindroth 1963) are united within the larger *Plataphus*-complex, even though in the latter two groups the anterior process of the metathorax is completely edged through a sharp arcuate impression in front of the apex, like the condition observed in numerous species from the *Ocydromus*-complex.

The basal edging of the elytra in *Bembidion ruruy* sp. n. appears to vary: in approximately half of the specimens from the type series it reaches the 4^th^ stria, versus the 5^th^ in the remaining individuals.

The differences in endophallus armament seem to be considerable when comparing *Bembidion ruruy* sp. n. to the description of *Bembidion ainu*. Regrettably, since the aedeagus sketch contained in the original description (Habu and Baba 1968: 146) fails to show a number of important details, it appears impossible to provide a thorough comparison between these two species, based on endophallus characters. Even though we are inclined to assign *Bembidion ruruy* sp. n. to the subgenus *Nipponobembidion* Habu & Baba, 1968, we prefer to question this placement now, because the diagnosis of that subgenus as formulated by Habu and Baba (1968) would otherwise require several amendments.

**Figure 7.**
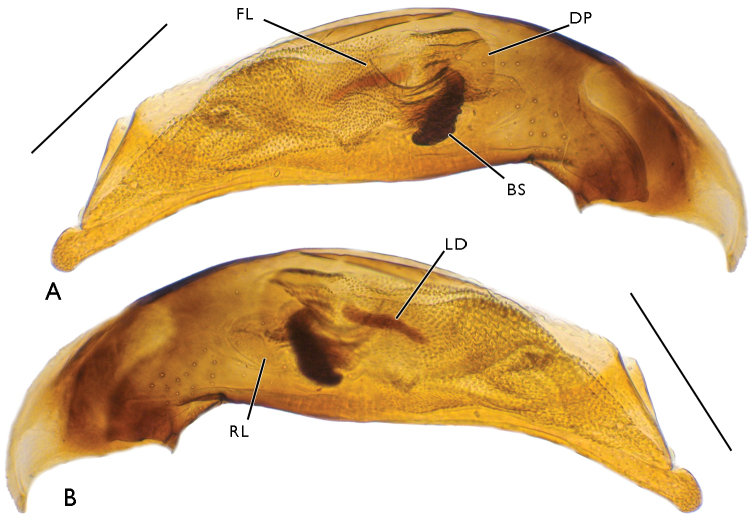
Aedeagus of Bembidion (Plataphodes) tetraporum Bates, 1883. Scale bars 0.25 mm. **A** right side **B** left side.

When describing *Bembidion ainu*, Habu and Baba (1968) noted that the length of the basal edging of the elytra, coupled with several other structural details, bring it closer to species of the subgenus *Plataphodes*. Based on a number of habitus features, as well as several other taxonomically important characteristics, the new species from Kunashir likewise seems to be similar to representatives of the subgenera *Plataphus* and *Plataphodes* in the sense of many authors (Netolitzky 1943, Habu 1973, Kryzhanovskij 1983, Kryzhanovskij et al. 1995, Marggi et al. 2003, Toledano 2008, 2009, Toledano and Nakládal 2011). The resemblance is particularly striking as regards the endophallus structure which *Bembidion ruruy* sp. n. shares with the syntopic *Bembidion tetraporum*: the central sclerite complex is nearly identical, a considerable difference being only observed in the length of the left laterodistal sclerite (Fig. 7).

Inasmuch as the main differences between *Nipponobembidion* and *Plataphodes* are related to the former’s wing reduction (and, consequently, shortened metepisterna and obliterated elytral humeri), also lying in body shape, *Nipponobembidion* seems best to be considered as a rather local and highly specialized derivative of *Plataphodes*. In assigning *Bembidion ruruy* sp. n. to *Nipponobembidion*, one must also consider its geographical proximity to *Bembidion ainu*, let alone the same geological and palaeogeographical background.

According to many authors (Velizhanin 1970, Briggs 1974, Korotkii et al. 1977, Korotkii 1985, Ota and Machida 1987, Levina and Grachev 1998, Bezverkhii et al. 2002, Pietsch et al. 2003, Bogatov et al. 2006, Vasilevskii 2008), during nearly the entire Pleistocene the Kunashir Island was connected to Hokkaido. Towards the Early Pleistocene (2.6 My), Sakhalin, Hokkaido and the southern Kuriles formed a large peninsula. The coastline of the Sea of Okhotsk in the Early Pleistocene (1.8 My) was close to the present-day one, all following changes in land area depending on climatic fluctuations.

A considerable sea transgression which was due to climate warming in the Late Pleistocene (130,000–70,000 y) separated Kunashir from Hokkaido, but the following two waves of cold (60,000–40,000 and 22,000–11,000 y) divided by a moderately warm climate resulted in a restored land connection of these two islands. In the Late Würm (15,000–13,000 y), another, rapid climate warming occurred, during which the rates of ice melting and sea-level rise dragged far behind climate change. The most intense interchanges of thermophilous biotic elements, especially those from Hokkaido to Kunashir, are believed to have taken place then, of course until the rising sea restored their isolation towards the mid-Holocene.

Pietsch et al. (2003), giving a biogeographical evaluation of the southern Kurile biota, noted that the modern flora and fauna of the islands are characterized by a high level of species diversity and a low degree of endemism. These authors considered this as an example of a “non-relict” genesis of the biome. The main roles in the formation of southern Kuriles’ biodiversity might have been played by migrations from Hokkaido. Even though during the entire Late Pleistocene no traces of a cover glaciation have been revealed, to a considerable degree the modern taxonomic composition of each of the island’s biota could have depended on the pioneer volcanic landscapes and on the presence of refugia on the volcanoes’ slopes in the form of thermal waters and fumarola fields (Razzhigaeva and Ganzei 2004). As a good example is the beetle Bembidion (Ocydromus) negrei Habu, 1958, known from several localities in Hokkaido and Honshu, but recorded on Kunashir Island only in thermal habitats in the caldera of Golovnin Volcano (Morita 2010, Ogawa 2014).

The above information concerning the morphological structure and geographical distribution of *Nipponobembidion*, coupled with the background geological and palaeoclimatic chronologies, suggests that this subgenus could have had a common ancestor with *Plataphodes*. Both currently known species of *Nipponobembidion* are only known from slopes of active volcanoes (Kimoto and Yasuda 1995, Masumoto 1980) showing fumarola and other thermal activities. Yet both differ considerably from each other, also suggesting their relict statuses. One may surmise that initially the ancestral form or forms could have been distributed over a single land mass composed of Sakhalin, Hokkaido and the southern Kuriles, with following cold climate phases reducing their ranges to one or a few fragments still confined to active volcanoes. It is remarkable that no *Nipponobembidion* has been found south of Hokkaido, albeit of Japan is fairly well known carabid fauna. This only supports our suggestion that thermal refugia may have played key roles in the formation of that subgenus. It seems as though, along with shrinking distributions, the ancestral populations could have switched to dwelling inside rocky and gravely grounds on warmed patches near fumarola fields and thermal water outlets. This might have been followed by wing reduction, shortened metepisterna, and rounded humeral angles of the elytra. The relatively small eyes developed as the result of a semi-endogean lifestyle might have led to a caudad shift of the posterior supra-ocular setae while flattened elytral striae to a reduced size of discal pores. To summarize, *Bembidion ruruy* sp. n. can be regarded as a relict element in the Kunashir fauna, most probably Late Pleistocene in age.

## Supplementary Material

XML Treatment for Bembidion (?Nipponobembidion) ruruy

## Supplementary materials

Supplementary material 1 Habitus of Bembidion (?Nipponobembidion) ruruy sp. n. female paratype, dorsal Data type: Image file. Explanation note: This image correspond to Fig. 1B in article. This dataset is made available under the Open Database License (http://opendatacommons.org/licenses/odbl/1.0/). The Open Database License (ODbL) is a license agreement intended to allow users to freely share, modify, and use this Dataset while maintaining this same freedom for others, provided that the original source and author(s) are credited.Kirill V. Makarov, Yuri N. Sundukov

Supplementary material 2 Habitus of Bembidion (?Nipponobembidion) ruruy sp. n. female paratype, ventral Data type: Image file. Explanation note: This image correspond to Fig. 1D in article. This dataset is made available under the Open Database License (http://opendatacommons.org/licenses/odbl/1.0/). The Open Database License (ODbL) is a license agreement intended to allow users to freely share, modify, and use this Dataset while maintaining this same freedom for others, provided that the original source and author(s) are credited.Kirill V. Makarov, Yuri N. Sundukov

Supplementary material 3 Habitus of Bembidion (?Nipponobembidion) ruruy sp. n. female paratype, lateral Data type: Image file. Explanation note: This image correspond to Fig. 3B in article. This dataset is made available under the Open Database License (http://opendatacommons.org/licenses/odbl/1.0/). The Open Database License (ODbL) is a license agreement intended to allow users to freely share, modify, and use this Dataset while maintaining this same freedom for others, provided that the original source and author(s) are credited.Kirill V. Makarov, Yuri N. Sundukov

Supplementary material 4 Habitus of Bembidion (?Nipponobembidion) ruruy sp. n. male holotype, dorsal Data type: Image file. Explanation note: This image correspond to Fig. 1A in article. This dataset is made available under the Open Database License (http://opendatacommons.org/licenses/odbl/1.0/). The Open Database License (ODbL) is a license agreement intended to allow users to freely share, modify, and use this Dataset while maintaining this same freedom for others, provided that the original source and author(s) are credited.Kirill V. Makarov, Yuri N. Sundukov

Supplementary material 5 Habitus of Bembidion (?Nipponobembidion) ruruy sp. n. male holotype, ventral Data type: Image file. Explanation note: This image correspond to Fig. 1C in article. This dataset is made available under the Open Database License (http://opendatacommons.org/licenses/odbl/1.0/). The Open Database License (ODbL) is a license agreement intended to allow users to freely share, modify, and use this Dataset while maintaining this same freedom for others, provided that the original source and author(s) are credited.Kirill V. Makarov, Yuri N. Sundukov

Supplementary material 6 Habitus of Bembidion (?Nipponobembidion) ruruy sp. n. male holotype, lateral Data type: Image file. Explanation note: This image correspond to Fig. 3A in article. This dataset is made available under the Open Database License (http://opendatacommons.org/licenses/odbl/1.0/). The Open Database License (ODbL) is a license agreement intended to allow users to freely share, modify, and use this Dataset while maintaining this same freedom for others, provided that the original source and author(s) are credited.Kirill V. Makarov, Yuri N. Sundukov

Supplementary material 7 Male genital apparatus of Bembidion (?Nipponobembidion) ruruy sp. n., holotype: aedeagus, left side Data type: Image file. Explanation note: This image correspond to Fig. 4B in article. This dataset is made available under the Open Database License (http://opendatacommons.org/licenses/odbl/1.0/). The Open Database License (ODbL) is a license agreement intended to allow users to freely share, modify, and use this Dataset while maintaining this same freedom for others, provided that the original source and author(s) are credited.Kirill V. Makarov, Yuri N. Sundukov

Supplementary material 8 Male genital apparatus of Bembidion (?Nipponobembidion) ruruy sp. n., holotype: aedeagus, right side Data type: Image file. Explanation note: This image correspond to Fig. 4A in article. This dataset is made available under the Open Database License (http://opendatacommons.org/licenses/odbl/1.0/). The Open Database License (ODbL) is a license agreement intended to allow users to freely share, modify, and use this Dataset while maintaining this same freedom for others, provided that the original source and author(s) are credited.Kirill V. Makarov, Yuri N. Sundukov
