# Supplementary figures and images for: Bembidion (?Nipponobembidion) ruruy sp. n., a new brachypterous ground beetle (Coleoptera, Carabidae) from Kunashir Island, Kuriles, Russia
Source: Zookeys. 2014 Dec 12;(463):75–93. doi: 10.3897/zookeys.463.8504 (PMC4294302; doi:10.3897/zookeys.463.8504)

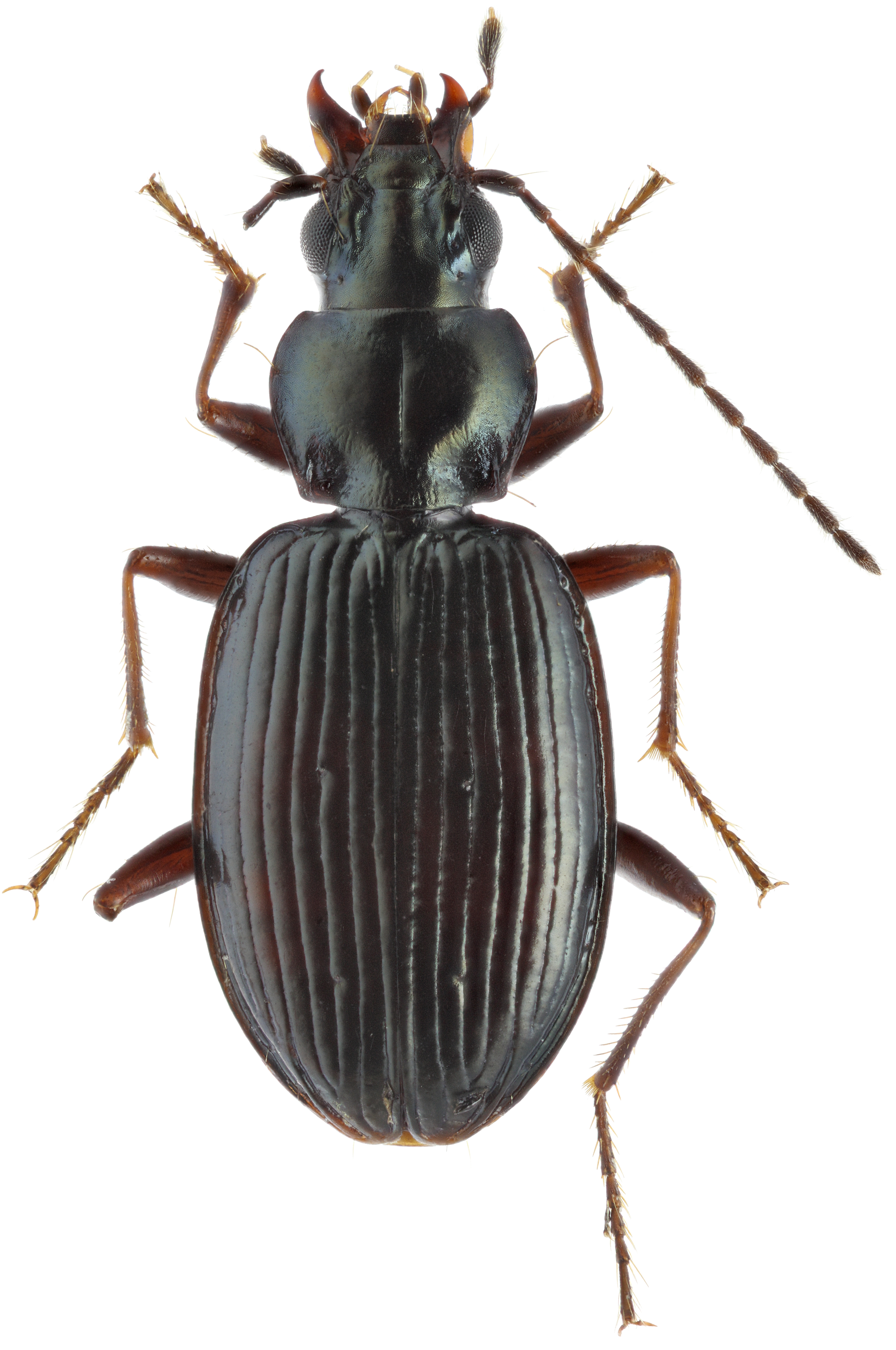

Supplement: Supplementary material 1 — Habitus of Bembidion (?Nipponobembidion) ruruy sp. n. female paratype, dorsal [file zookeys-463-075-s001.jpg]

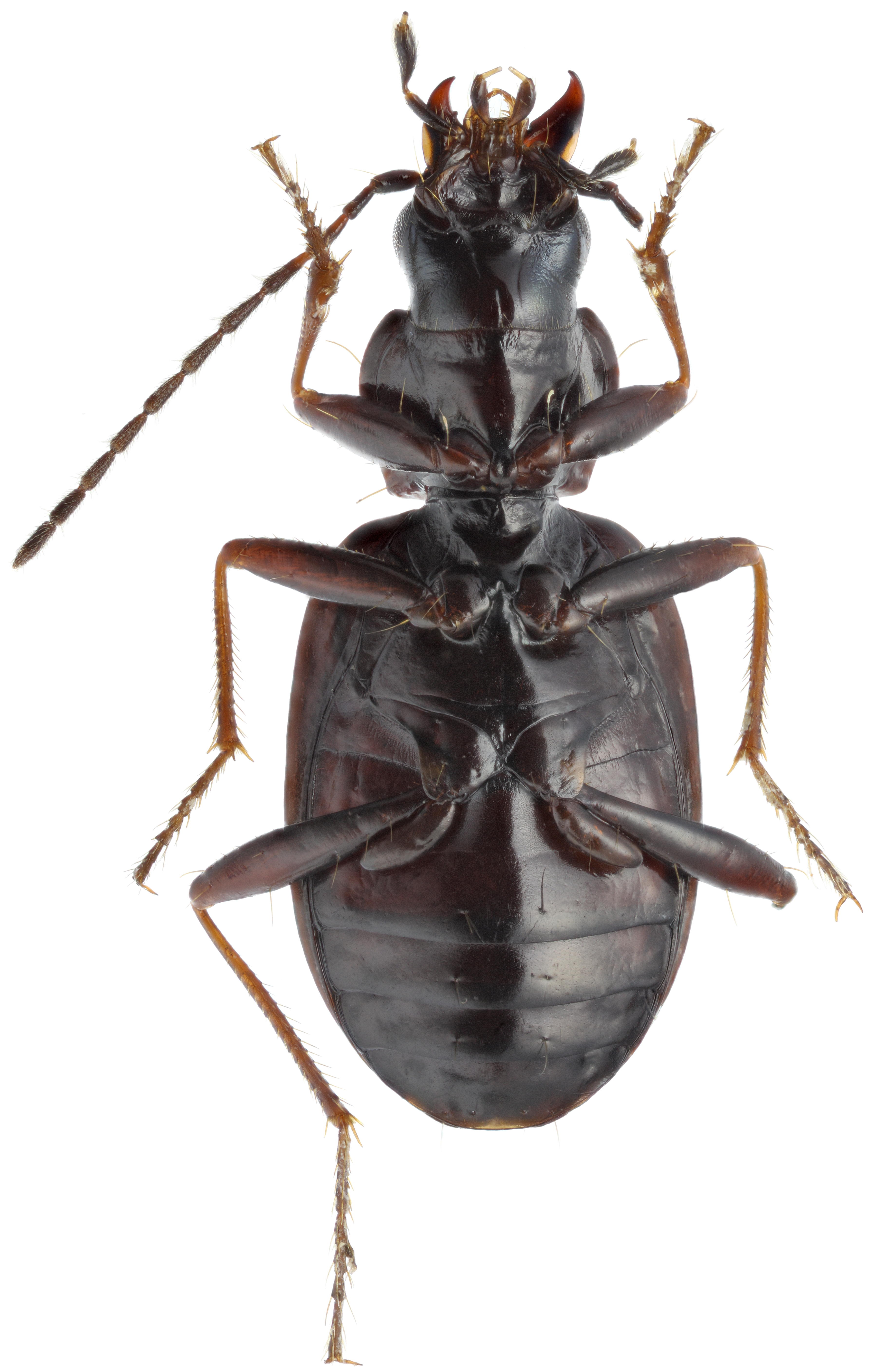

Supplement: Supplementary material 2 — Habitus of Bembidion (?Nipponobembidion) ruruy sp. n. female paratype, ventral [file zookeys-463-075-s002.jpg]

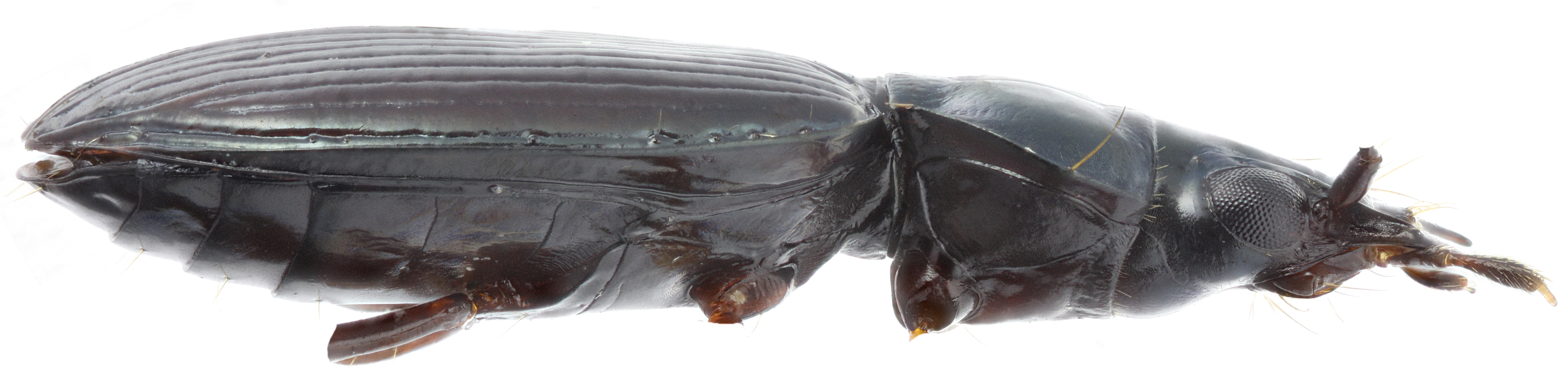

Supplement: Supplementary material 3 — Habitus of Bembidion (?Nipponobembidion) ruruy sp. n. female paratype, lateral [file zookeys-463-075-s003.jpg]

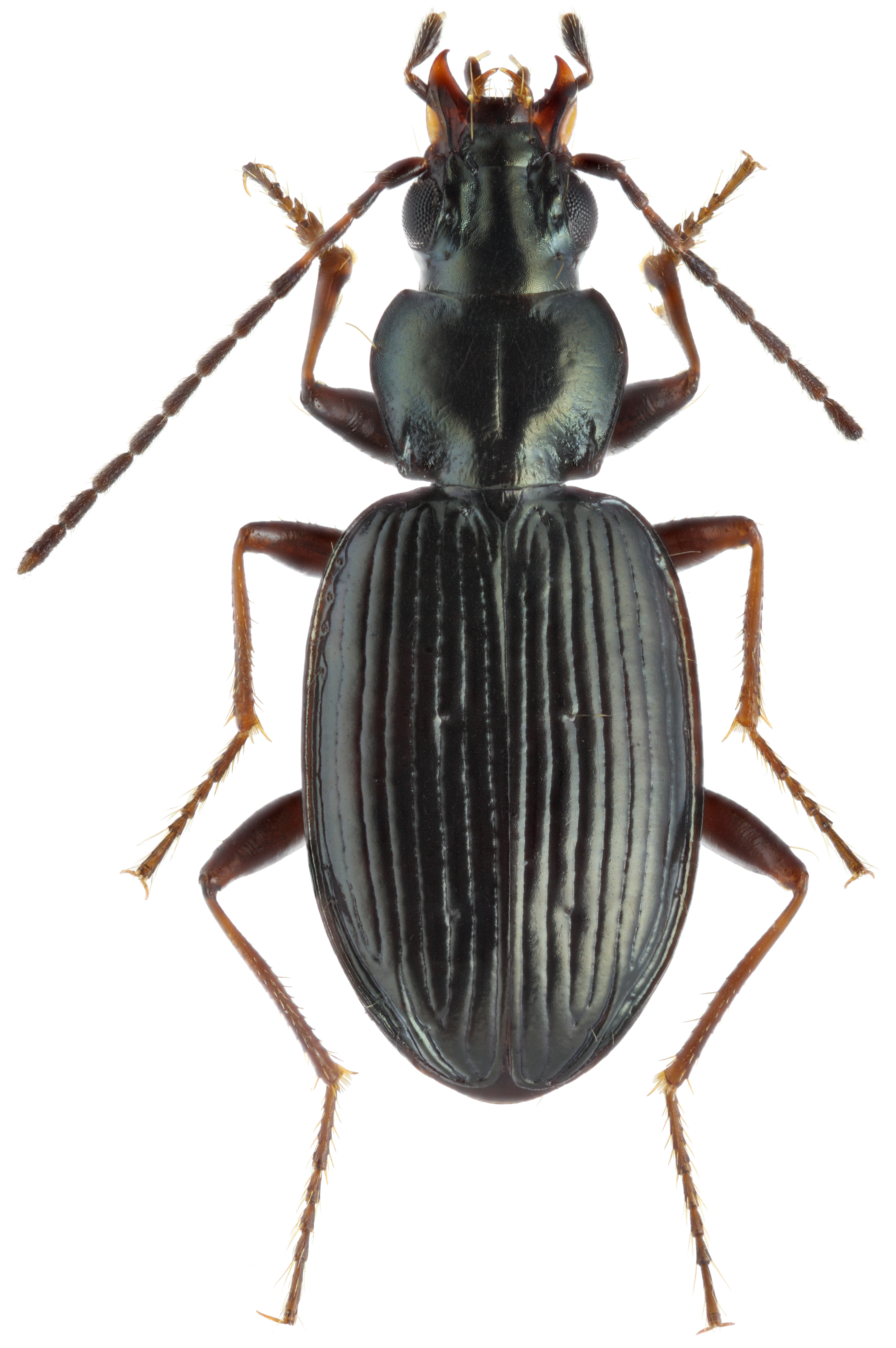

Supplement: Supplementary material 4 — Habitus of Bembidion (?Nipponobembidion) ruruy sp. n. male holotype, dorsal [file zookeys-463-075-s004.jpg]

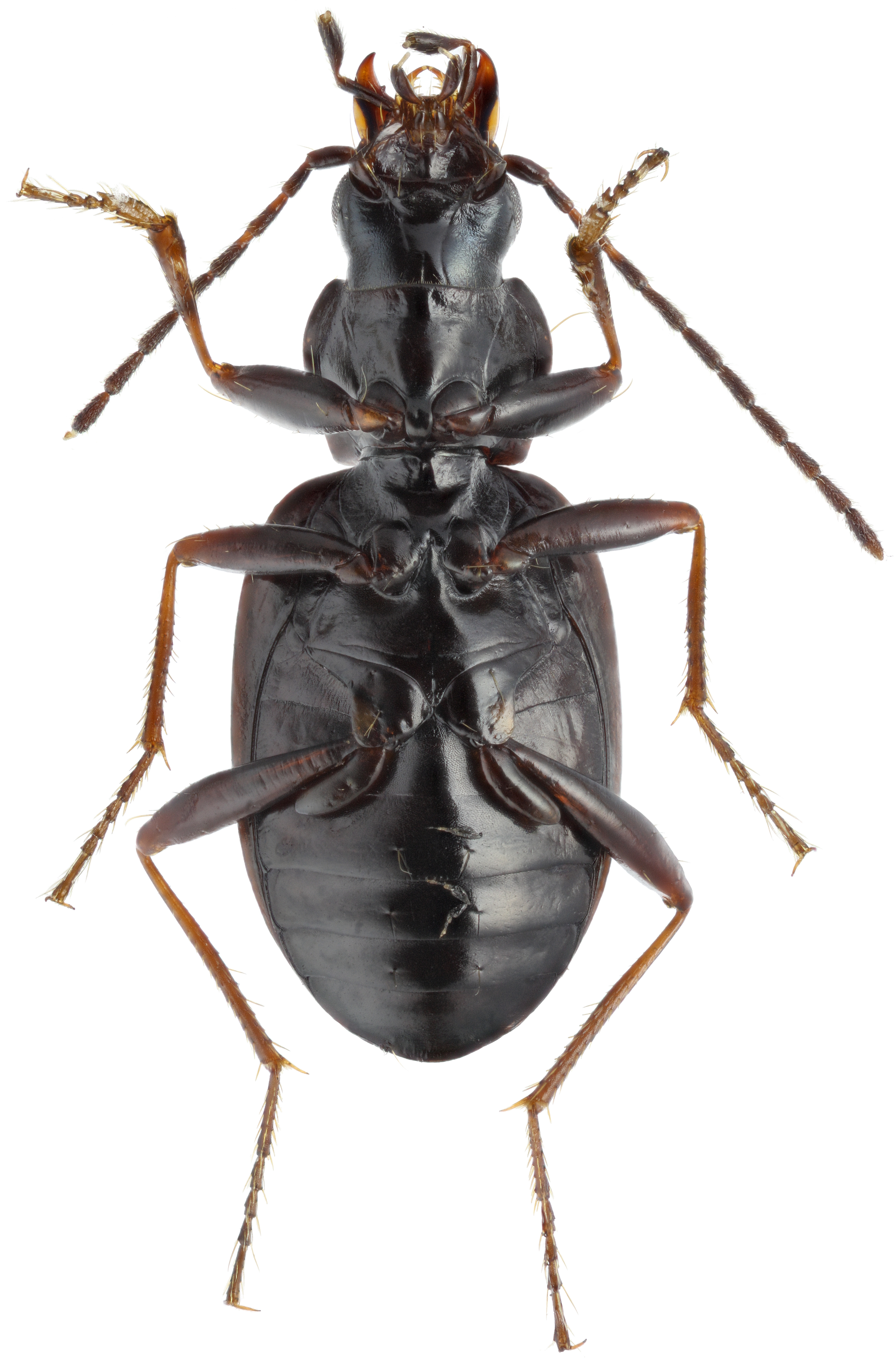

Supplement: Supplementary material 5 — Habitus of Bembidion (?Nipponobembidion) ruruy sp. n. male holotype, ventral [file zookeys-463-075-s005.jpg]

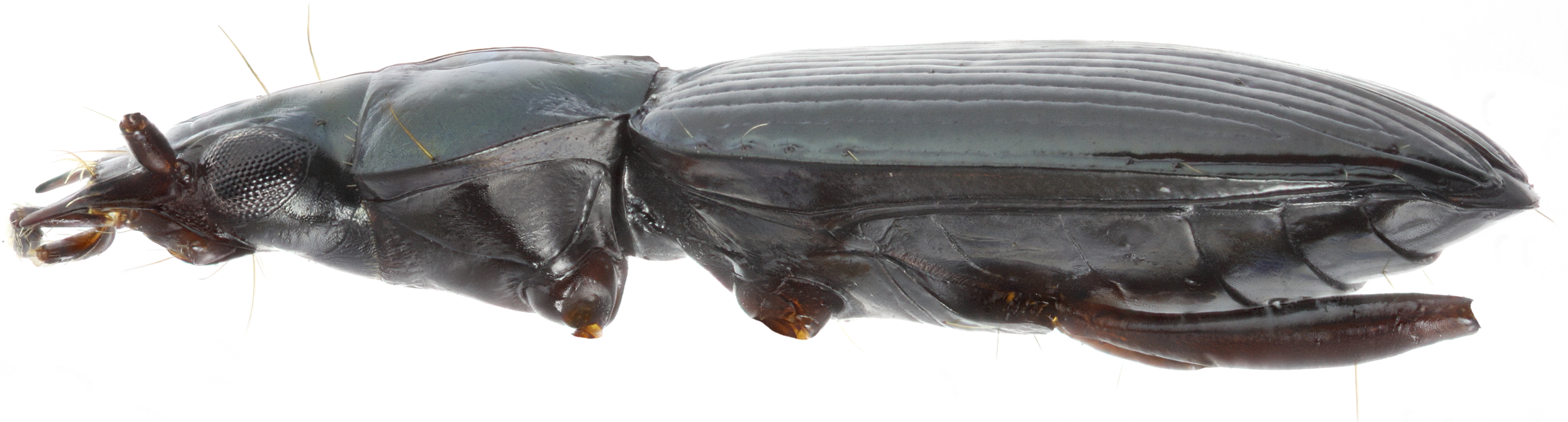

Supplement: Supplementary material 6 — Habitus of Bembidion (?Nipponobembidion) ruruy sp. n. male holotype, lateral [file zookeys-463-075-s006.jpg]

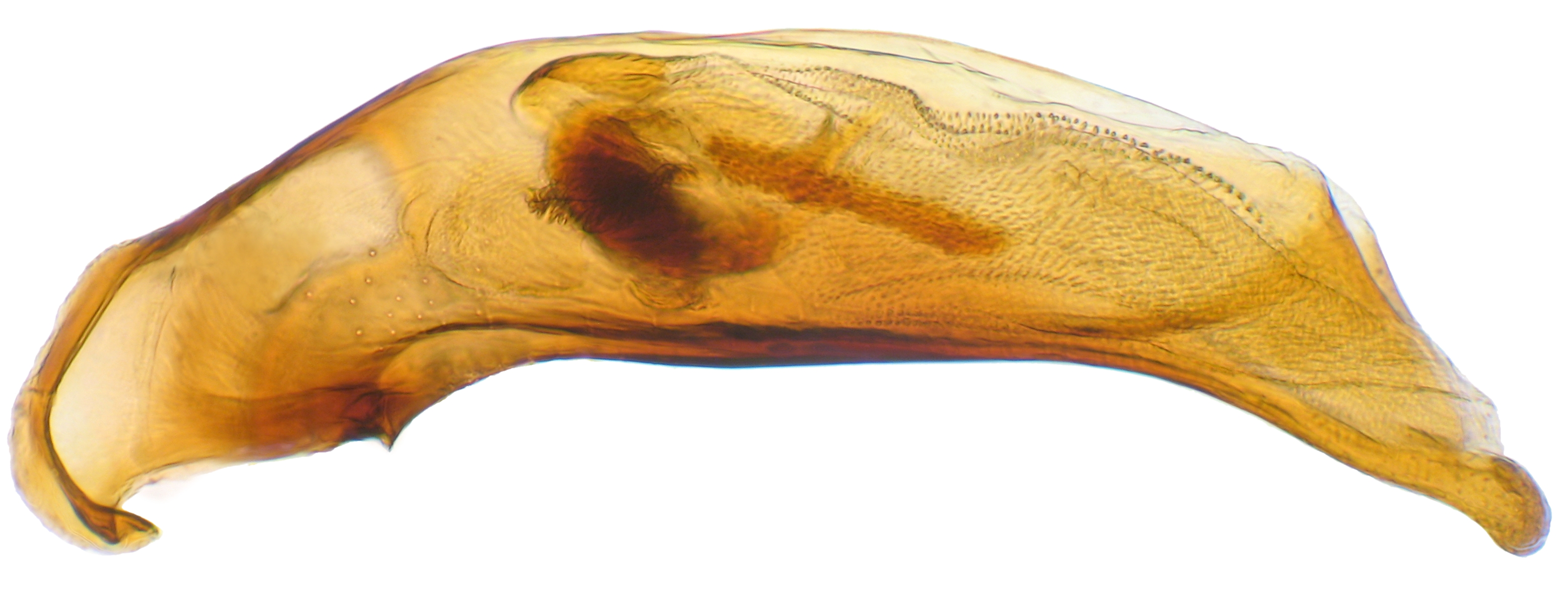

Supplement: Supplementary material 7 — Male genital apparatus of Bembidion (?Nipponobembidion) ruruy sp. n., holotype: aedeagus, left side [file zookeys-463-075-s007.jpg]

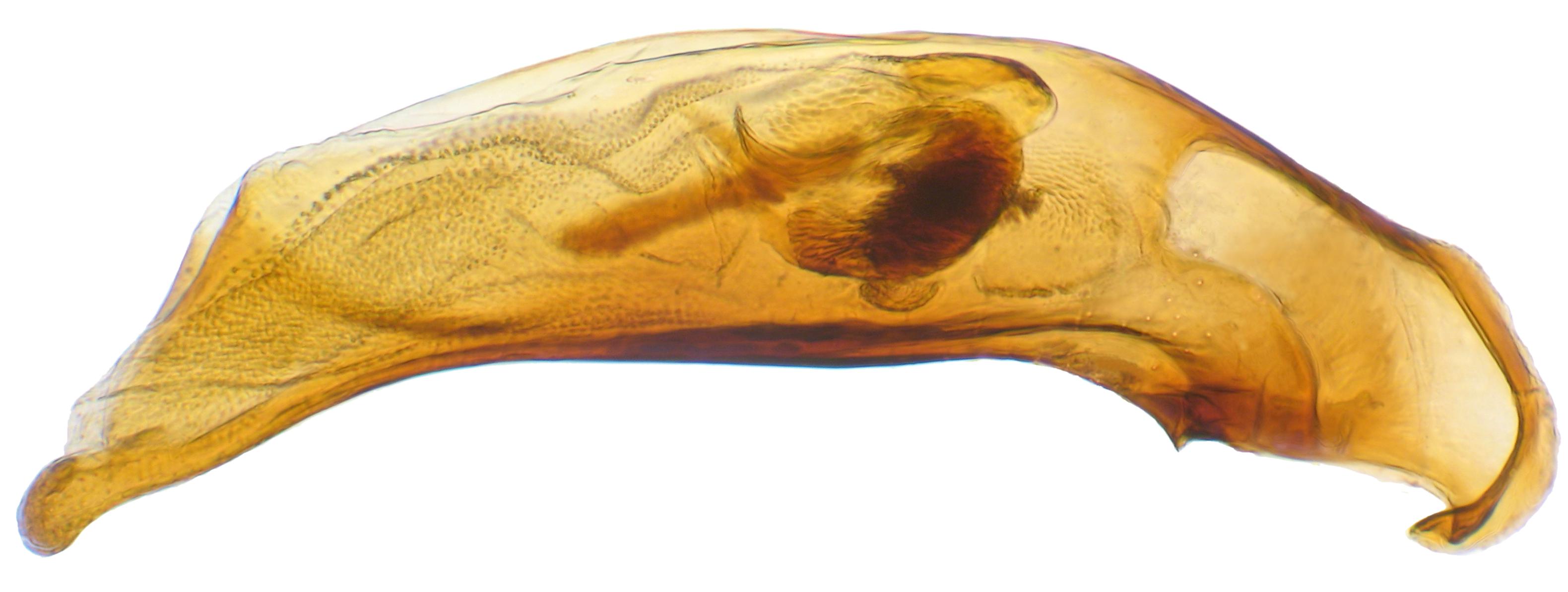

Supplement: Supplementary material 8 — Male genital apparatus of Bembidion (?Nipponobembidion) ruruy sp. n., holotype: aedeagus, right side [file zookeys-463-075-s008.jpg]
